# Independence in Medical Journalism

**Published:** 1908-01

**Authors:** 


### "INDEPENDENCE" IN MEDICAL JOURNALISM.

———

The *West Virginia Medical Journal* for June, in an article entitled "Independent" Journalism, takes as its text a commendatory notice of itself in the Saint Louis Medical Review for February 9th. In this article, the *Journal* takes exception to the general use of the term "independent," viz., as distinguishing privately owned journals from the official organs of professional bodies, and argues that "the conditions necessary to constitute independence in medical journalism are much more likely to be found when the journals are conducted by State or society organizations that have no connection with business enterprises, or have no dependence for their continuance on the financial support derived from subscriptions and advertising patronage."

We may point out *en passant* that the State journal is practically as much dependent "on subscriptions" as the so-called independent journal. If this is doubted, let the said State journal editors adopt a policy which will be displeasing to their clientele, the State organization, and watch the result. Moreover, as few of them have any circulation to speak of outside of the organization that maintains them, if the members of that organization—upon which it is conceded they depend for their maintenance—are *not* "subscribers," we would suggest that they call the attention of the postal authorities to the fact and see what will happen. It is not true, therefore, to say that they are not dependent on their subscribers. They are just as much so, if not rather more, than any other kind of journal, and their circulation usually evidences the fact in a somewhat backhanded manner.

But our purpose was not to deal with details, but to examine general principles. And right at the beginning we join issue with our contemporary as to what constitutes "independence" in journalism. Our contemporary appears to assume that nothing can display independence of spirit that has to look to others for sustenance. This idea is contradicted every day when we find true independence often displayed by those dependent on others.

for subsistence, and servility *ad nauseam* on the part of others who could well afford to have the courage of their convictions on any subject whatever. As a matter of fact, this question of the source of finances—whether from subscriptions or advertising—is only a very small factor in the question of that control likely to prove detrimental to true independence, which is not a simple question relating to advertising matter, but covers a much wider field.

To be truly independent, a journal must, in our opinion, refuse to be limited by orthodoxy. It must accord to heresy a right of free expression, so long as the evidence adduced is not a mere repetition of already disproved allegations or confuted arguments, the language is temperate, and the writer shows acquaintance with the subject as a whole, an appreciation of the position attacked as it is seen from the orthodox viewpoint, and not merely in the distorted vision of an "anti." Nay more, it must be prepared to follow "heresy" and become a "heretic" if convinced.

It must also repudiate the cardinal idea once forcibly expressed in a controversy that we well remember, in the words, "on this subject the profession has long since spoken definitively." The profession can not speak definitively on any subject at any time. It may think it has done so, but its own history and that of all science amply demonstrates that there can be no such thing as definitiveness in the empirical (or inductive) sciences, since their rulings must always be subject to reversal by new findings until the human race shall have attained to infinite intelligence.

These two factors are as important as, if not more important than, the freedom from a commercial bias in regard to a small section of the subjects dealt with by professional journals, viz., that of materia medica, of which alone our contemporary appears to take note.

Now, with regard to the first two of these points, we have no hesitation in asserting that from the very nature of things the organization journal must find it very much harder to be truly independent, than does any privately owned journal—with all its alleged "subsidizing" by proprietary medicine makers—in regard to the materia medica. Indeed, on the first of them, it has absolutely no right to be independent, for it is the organ of the majority, pledged to their views, a bounden partisan of their orthodoxy. Even if it had a right, it is quite certain that should it commit itself to what is generally held to be heresy, the majority of its constituents would compel its editor either to recant—no matter what his inner convictions might be—or to resign in favor of some one more orthodox. Yet the merest glimpse at the history of science, and particularly of medicine, will abundantly demonstrate that many of the most violently opposed heresies of the past, opposed even to the point of bitter persecution, are today among the most orthodox of views. We need only instance Semmelweis and the doctrine of puerperal fever. Had Semmelweis been the editor of an organization journal, how long would he have held office? Where is the independence in that? How many editors of organization journals would dare, though their convictions led them that way, to challenge the mosquito theory of malaria and yellow fever, to repudiate the propriety of compulsory vaccination, even to deny the usefulness of vaccination or diphtheria antitoxin? And if they did, how long would any of them remain editors? We believe in all these things, it is true; but if we did not, if evidence appeared to us forthcoming cogent enough to make us see that in these instances (as in many others beside that of the nature of puerperal fever on account of which the profession hounded Semmelweis to his death and later erected a monument to him in recognition of his discovery) the "definitive" decree of the profession was wrong, we should not hesitate to say so and no one could depose us from our chair. The worst that could happen to the independent journal under such circumstances would be that individual subscribers, few or many, who felt sufficiently aggrieved shoul withdraw their subscriptions. Not so with the organization journal. Its editor would have put every member of his organization who did not agree with him, as well as the organization as a whole, in a false position, as appearing to espouse a doctrine considered by them, individually and collectively, false.

The history of Semmelweis, and innumerable instances, only go to show that it would be the blackest of days for medicine should the journal independent of orthodox absolute control ever be blotted out.—Ed., St. Louis Medical Review, Sept. 1907.

